# Supplementary material for: Dual nature of magnetic nanoparticle dispersions enables control over short-range attraction and long-range repulsion interactions
Source: Commun Chem. 2022 Jun 9;5:72. doi: 10.1038/s42004-022-00687-3 (PMC9814898; doi:10.1038/s42004-022-00687-3)
Supplement: Supplementary file 2 — Supplementary Information [file 42004_2022_687_MOESM2_ESM.pdf]

*Supplementary Materials*

# Dual nature of magnetic nanoparticle dispersions enables control over short-range attraction and long-range repulsion interactions

Ahmed Al Harraq[1], Aubry A. Hymel[1], Emily Lin[2], Thomas M. Truskett[2], Bhuvnesh Bharti[1]*
[1]Cain Department of Chemical Engineering, Louisiana State University, Baton Rouge, Louisiana 70803, USA
[2]McKetta Department of Chemical Engineering, University of Texas at Austin, Austin, Texas 78712, USA
*Corresponding author. Email: bbharti@lsu.edu

**Supplementary Note 1**

DLVO interactions

The Derjaguin-Landau-Verwey-Overbeek (DLVO) interaction energy, $U_{\mathrm{DLVO}}$, is the sum of the electrostatic and van der Waals interaction energies $U_{\mathrm{e}}$ and $U_{\mathrm{vdW}}$, respectively.[32]

$$U_{\mathrm{DLVO}}(D) = U_{\mathrm{e}} + U_{\mathrm{vdW}} \tag{1}$$

where D is the surface-to-surface distance between particles.

The electrostatic interaction between particles of radius *R* is given by the following expression:

$$U_{\mathrm{e}} = \frac{R}{2} Z\, e^{-\kappa D} \tag{2}$$

where $\kappa$ is the Hückel parameter, i.e., the inverse of the Debye length and *Z* is a factor expressed as follows:

$$Z = 64\pi\varepsilon_0\varepsilon \left(\frac{k\mathrm{T}}{e}\right)^2 \tanh^2\left(\frac{eP}{4kT}\right) \tag{3}$$

In Equation (3), $\varepsilon_0$ and $\varepsilon$ are respectively the permittivity of free space and of the medium, *k* is Boltzmann's constant, T is the system temperature, *e* is the elementary charge of an electron and *P* is the surface potential of the particles approximated as the measured zeta potential.

The van der Waals interactions are calculated from the following equation:

$$U_{\mathrm{vdW}} = -\frac{AR}{12D} \tag{4}$$

where *A* is the Hamaker constant of polystyrene colloids.
The partial overlap of counter ion double layers of negatively charged particles leads to an osmotic pressure and repulsion between interacting particles. Such repulsion impedes the irreversible aggregation of particles and explains their observed stability in suspension, in the absence of nanoparticles.

## Supplementary figures

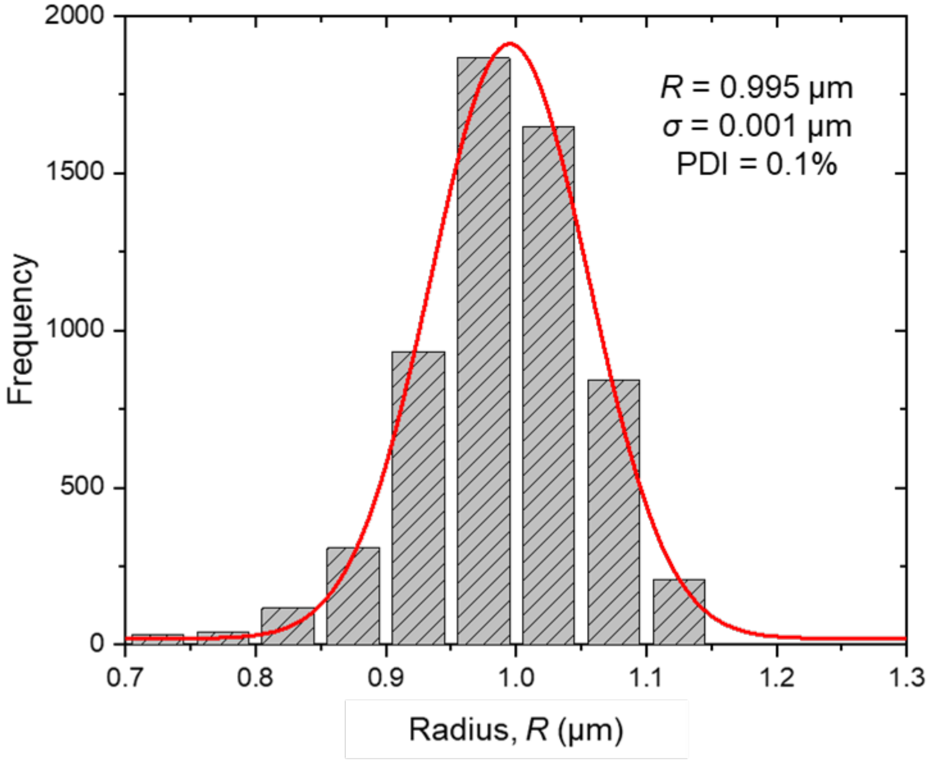


**Supplementary Figure 1.**
Size distribution of polystyrene microparticles as obtained by image analysis. The distribution yields a mean value of $R$ = 1.0 μm equivalent to a polydispersity index (PDI) of 0.1%. Here σ is the standard deviation of the distribution.

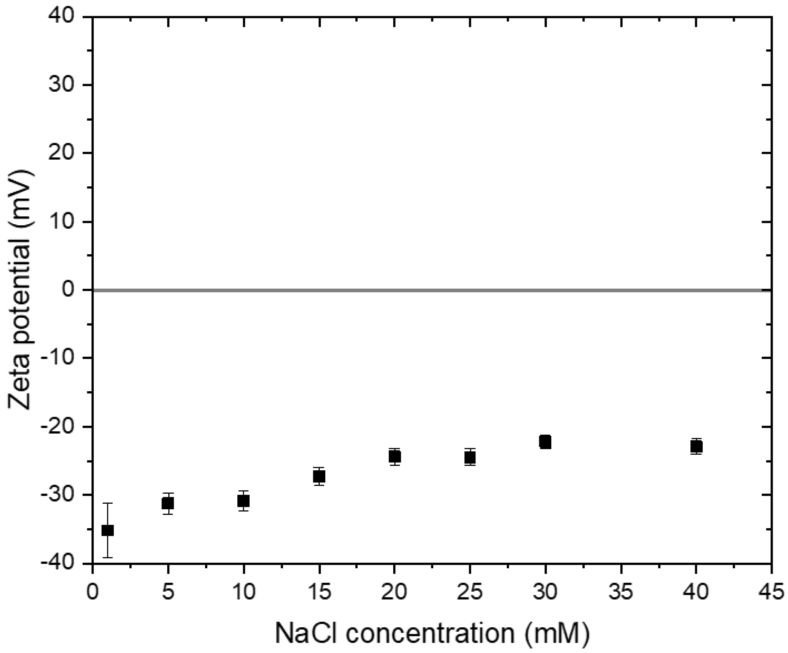


**Supplementary Figure 2.**

Measurement of the zeta potential of polystyrene microparticles as a function of salt concentration. The zeta potential is highly negative at low salt concentration indicating electrostatic repulsion between particles. This is attenuated with increasing ionic concentration which has the effect of 'thinning' the electric double layer surrounding particles. Measurements were done using the Litesizer 500 from Anton Paar, with the Univette setup.

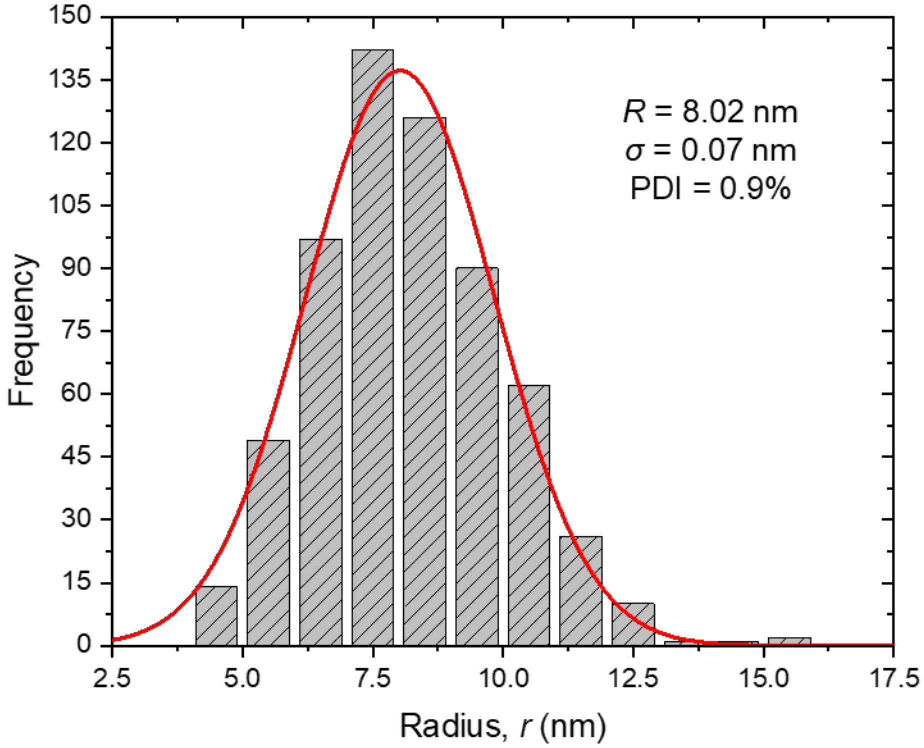


**Supplementary Figure 3.**

Size distribution of iron oxide nanoparticles from EMG 705, obtained from image analysis of multiple transmission electron micrographs. The distribution yields a mean value of $r$ = 8.0 nm with a polydispersity index (PDI) of 0.9%. Here σ is the standard deviation of the distribution.

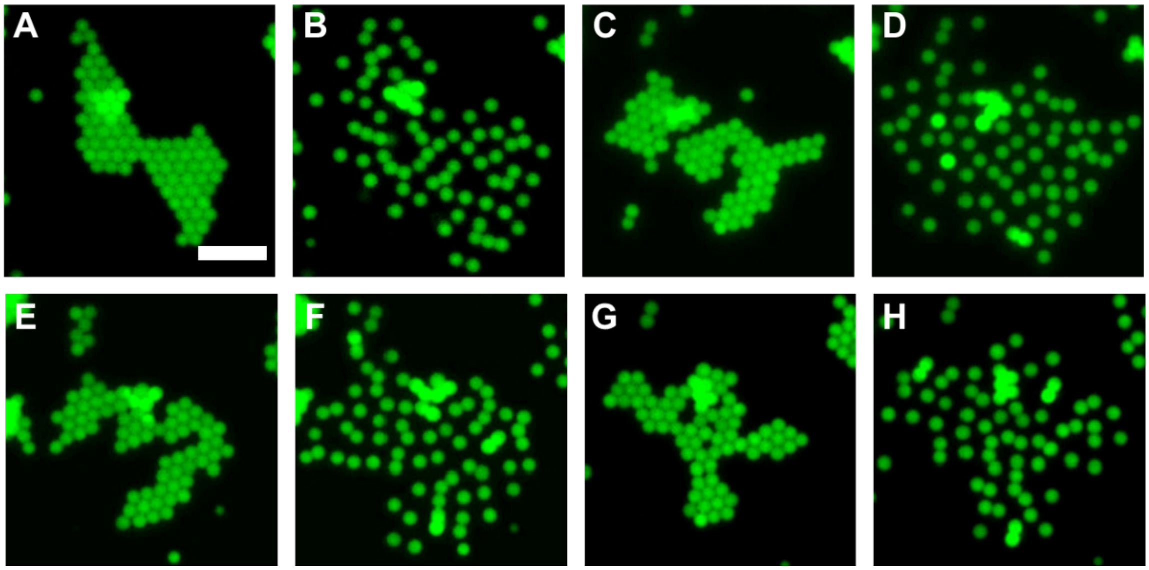


**Supplementary Figure 4.**
Time sequence of assembly and disassembly of crystallites from Movie S2. Each assembled state is disassembled in <10 seconds at $\boldsymbol{H}$ = 1000 A $m^{-1}$ and reassembly is allowed to proceed for 1 hour at $\boldsymbol{H}$ = 0 A $m^{-1}$.

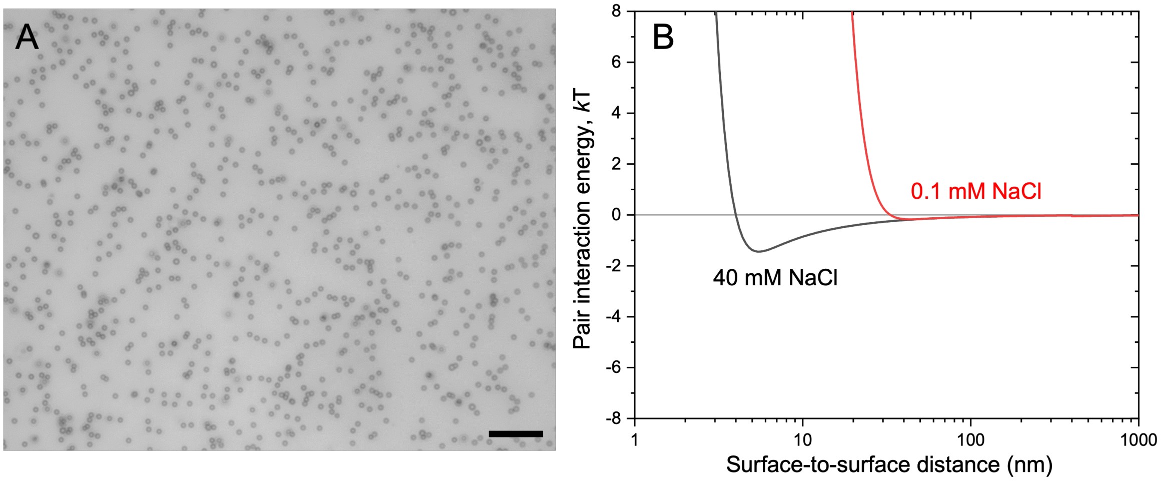


**Supplementary Figure 5.**
(A) Micrograph of particles suspended for more than 1 hour in 40 mM NaCl solution, without any nanoparticle depletant. Scale bar: 20 µm. (B) Plots of DLVO interaction between PS microparticles without the addition of salt (red line) and in 40 mM NaCl solution (black line). Adding electrolyte screens electrostatic double layer repulsion to subsequently enable the short-range excluded volume effects of the depletant.

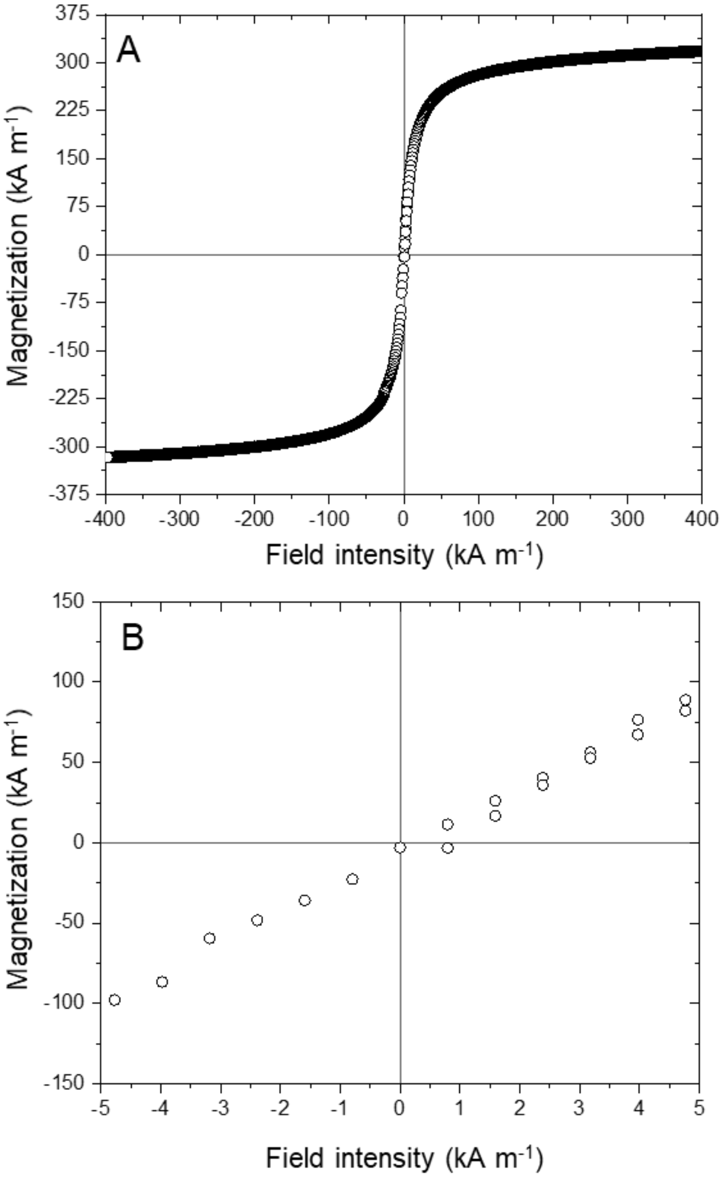


**Supplementary Figure 6.**
Superconducting Quantum Interference Device (SQUID) magnetometry measurement for ferrofluid. *(A)* The magnetization curve shows saturation at fields approximately 50,000 A $m^{-1}$ in magnitude and no hysteresis. *(B)* Ferrofluid magnetization is linear in the region of operational use: the slope of the line indicates a bulk magnetic susceptibility of ~20.

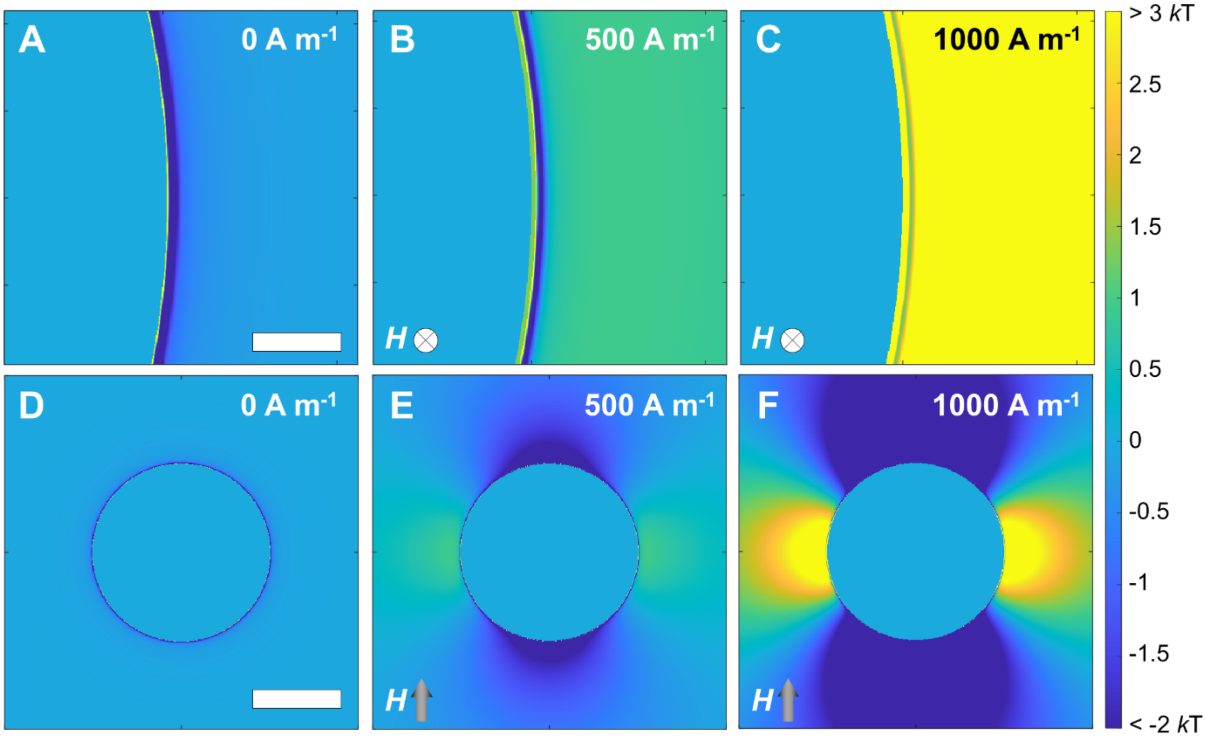


**Supplementary Figure 7.**
2D color maps of net pair interaction energy surrounding a single microsphere. (A-C) Close-up on microsphere surface showing the unhindered short-range attraction at ***H*** = 0 A m$^{-1}$, the strong long-range repulsion at ***H*** = 1000 A m$^{-1}$, and the competition between short-range attraction and long-range repulsion at ***H*** = 500 A m$^{-1}$. Scale bar: 100 nm. (D-F) Pair interaction energy landscapes equivalent to those shown in Fig. 3D-F but computed with an in-plane magnetic field. This highlights the anisotropic nature of the magnetic interaction which is both attractive in the direction parallel to ***H*** and repulsive in the direction orthogonal to ***H***.

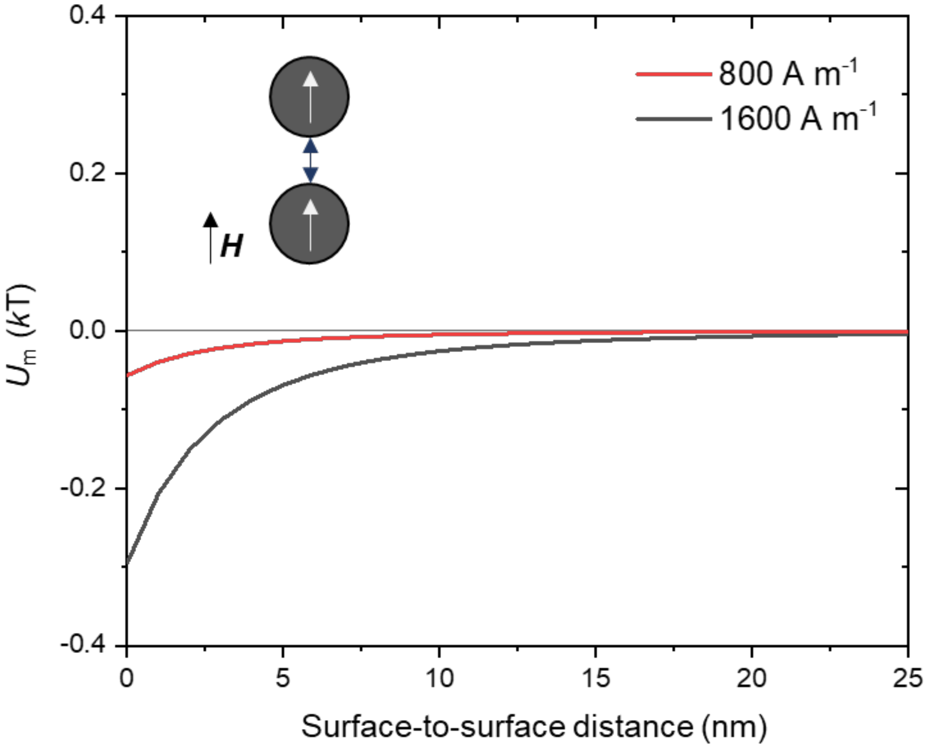


**Supplementary Figure 8.**
Magnetic dipolar interaction energy between two $Fe_3O_4$ nanoparticles estimated from measured moments at field strengths of 800 A m$^{-1}$ and 1600 A m$^{-1}$, using point dipole approximation The dipolar attraction between nanoparticles in the range of external field strengths used in experiments is insufficient to induce assembly.

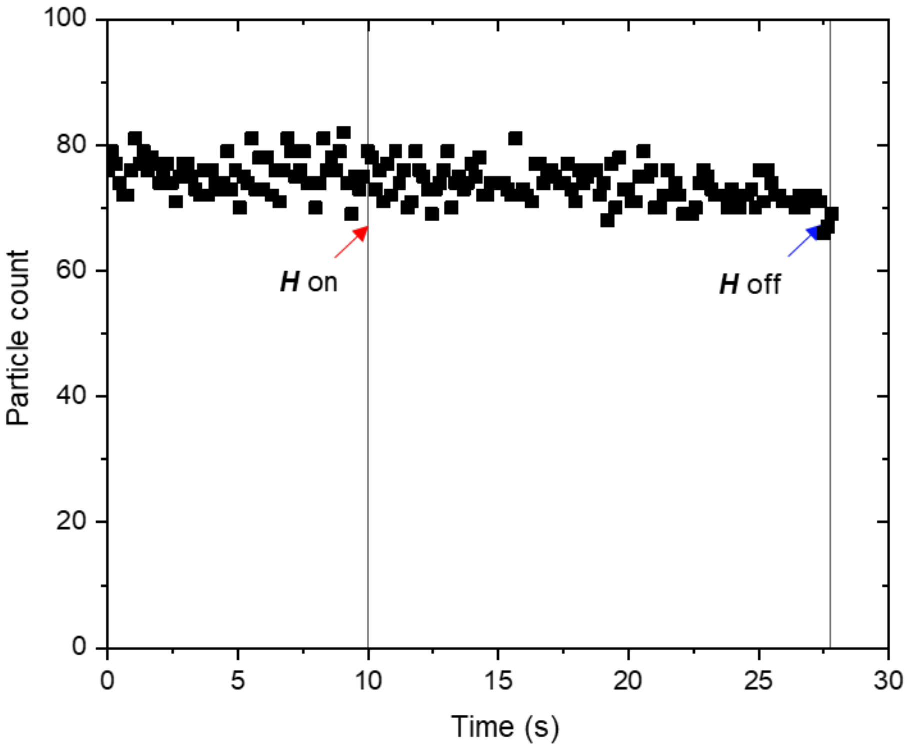


**Supplementary Figure 9.**
Change in the number of particles observed when disassembling a crystal with the external magnetic field at $\boldsymbol{H} = 970$ A m$^{-1}$. The count obtained analyzing each frame indicates minimal transition from a 2D crystal to a chain-like configuration (aligned orthogonal to viewing plane) during the field exposure.

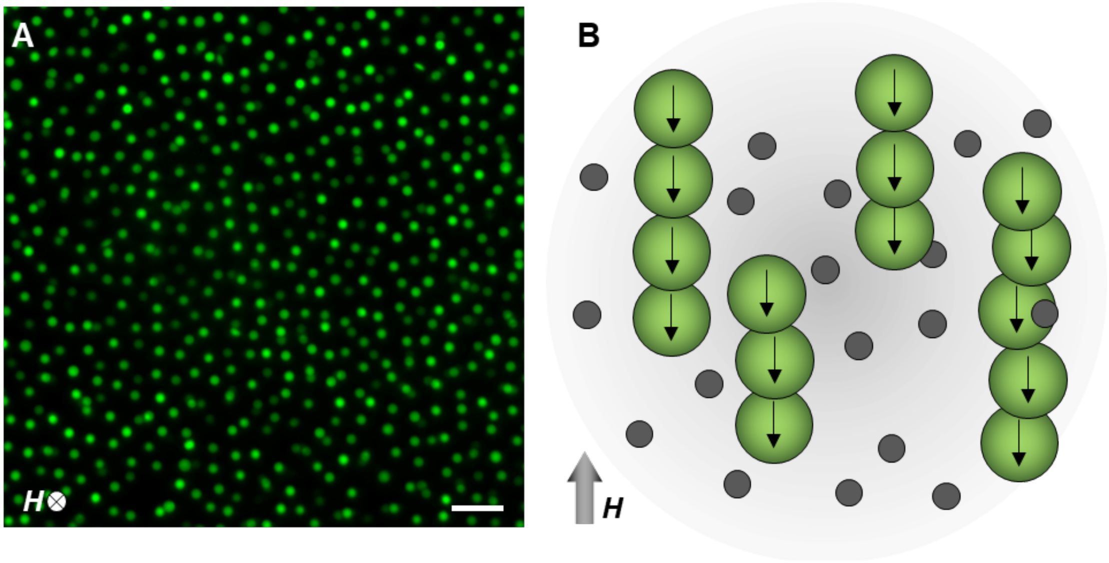


**Supplementary Figure 10.** (A) Fluorescence micrograph and (B) schematic of out-of-plane chaining of microparticles observed when applying a magnetic field with ***H*** = 2000 A m$^{-1}$. Scale bar: 10 μm.

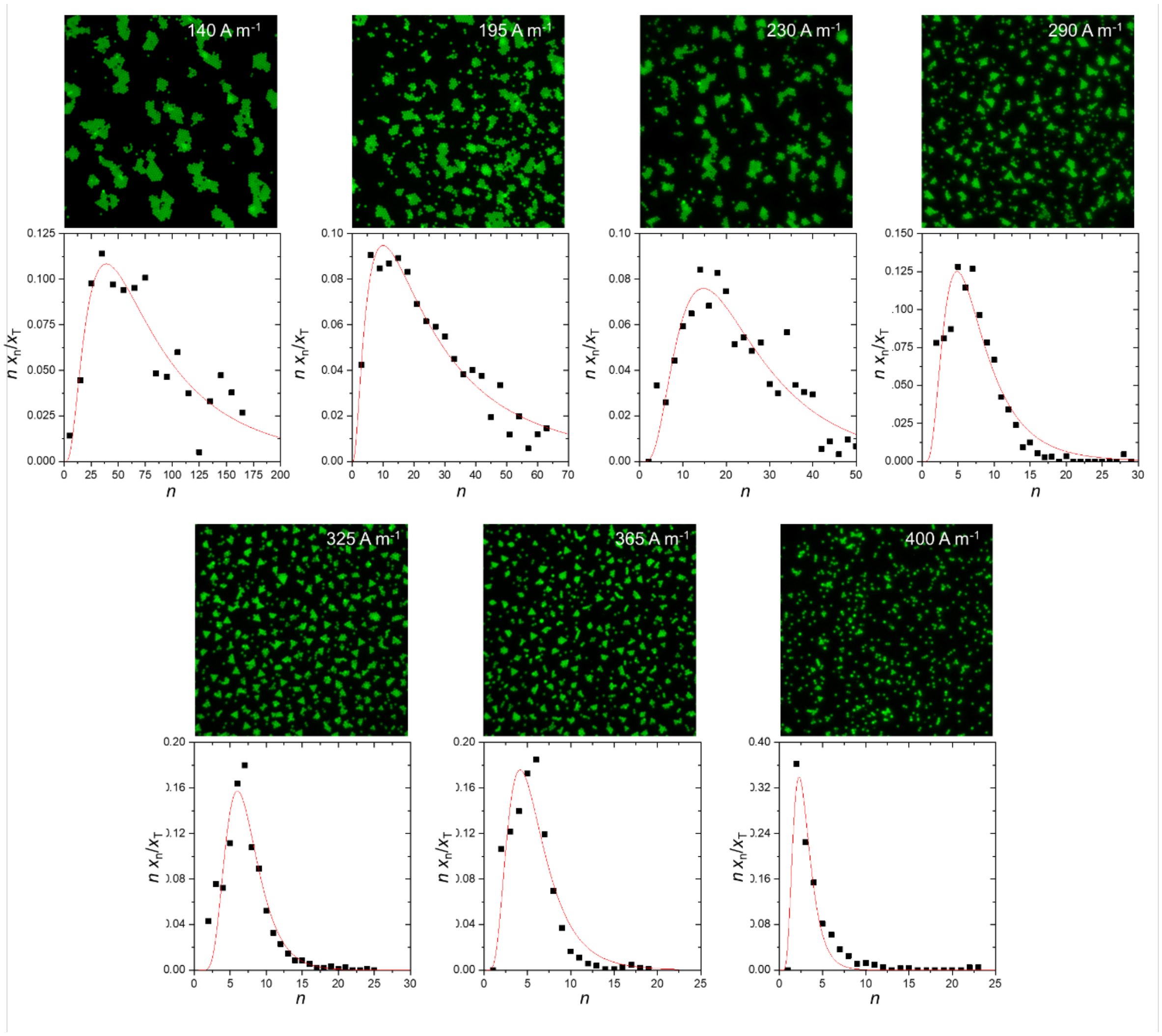


**Supplementary Figure 11.**
Fluorescence micrographs of clusters formed after ~12 hours of exposure to the external magnetic field of increasing strength. The size distribution of clusters is computed from the number $x_n$ of particles in clusters of size $n$ divided by the total number of particles counted $x_T$. The red lines are the log-normal fit to the experimental points.

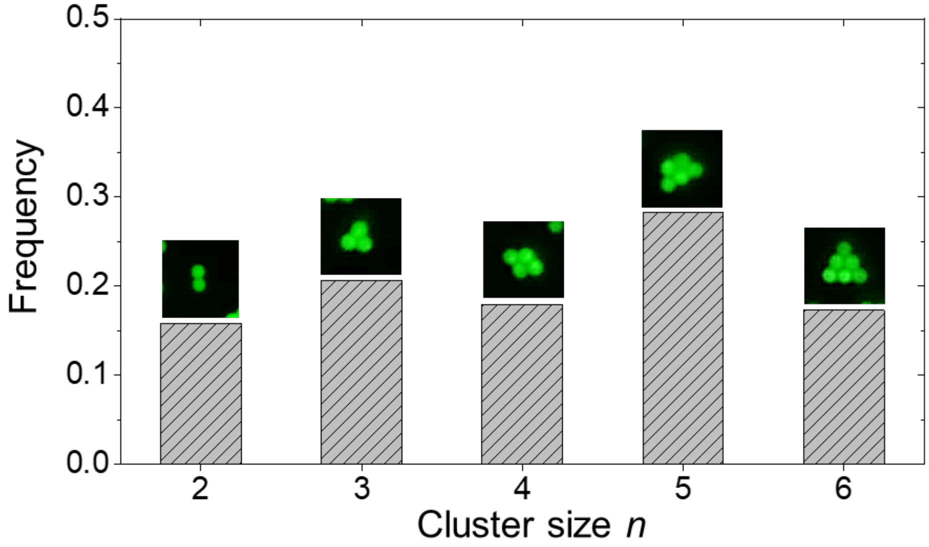


**Supplementary Figure 12.**
Sample distribution of assembled clusters from $n$ = 2 to $n$ = 6 obtained after 12 hours of field exposure at $\boldsymbol{H}$ = 365 A m$^{-1}$.

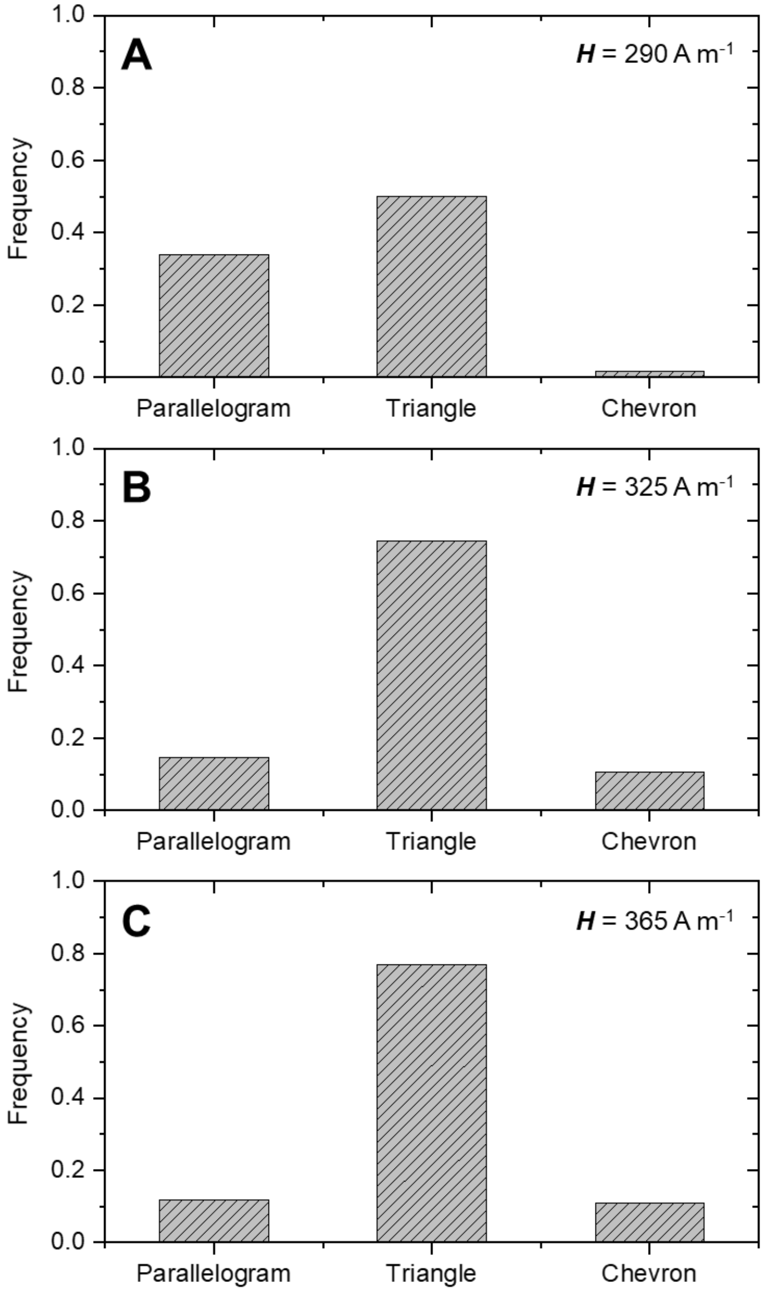


**Supplementary Figure 13.**
Frequency of occurrence of parallelogram, triangle and chevron configurations of hexamers for (A) $\boldsymbol{H}$ = 290 A m$^{-1}$, (B) $\boldsymbol{H}$ = 325 A m$^{-1}$, and (C) $\boldsymbol{H}$ = 365 A m$^{-1}$.

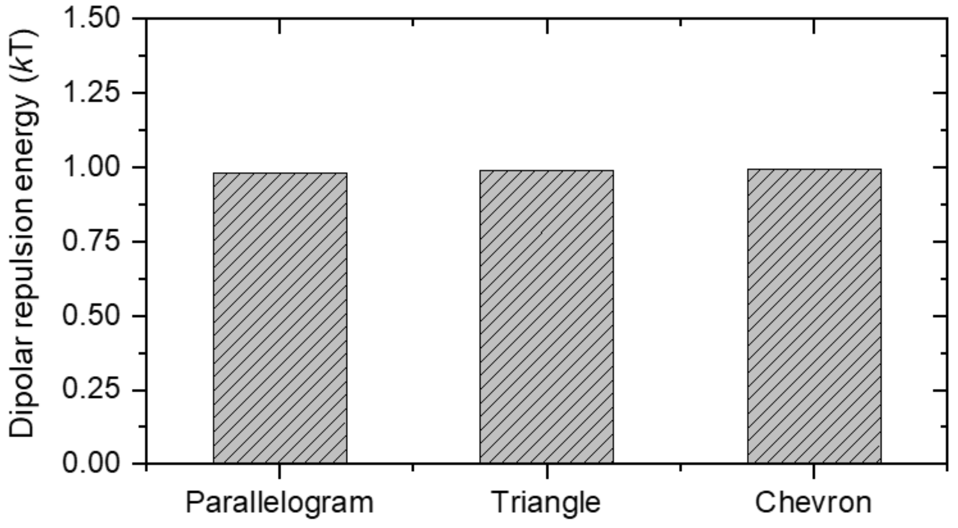


**Supplementary Figure 14.**
Calculated dipolar repulsion energy of 6-particle clusters in the parallelogram, triangle, and chevron configurations. Values are obtained by adding up all pairwise repulsion among all particles in the clusters and dividing the total by 6.

| Field intensity ***H*** | Mean cluster size *n* | Standard deviation σ | Particle count |
|---|---|---|---|
| 135 A $m^{-1}$ | 73.18 | 7.33 | 24560 |
| 195 A $m^{-1}$ | 25.03 | 1.55 | 30072 |
| 230 A $m^{-1}$ | 22.03 | 1.17 | 17799 |
| 290 A $m^{-1}$ | 6.82 | 0.29 | 5964 |
| 325 A $m^{-1}$ | 6.90 | 0.22 | 17799 |
| 365 A $m^{-1}$ | 5.36 | 0.28 | 18817 |
| 400 A $m^{-1}$ | 2.73 | 0.09 | 4790 |

**Supplementary Table 1.**

Values of mean cluster size $\bar{n}$ with associated standard deviation and standard error resulting from the log-normal distribution of cluster sized obtained via image analysis.
