# Supplementary material for: Dual nature of magnetic nanoparticle dispersions enables control over short-range attraction and long-range repulsion interactions
Source: Commun Chem. 2022 Jun 9;5:72. doi: 10.1038/s42004-022-00687-3 (PMC9814898; doi:10.1038/s42004-022-00687-3)
Supplement: Supplementary file 3 — Decription of Supplementary files [file 42004_2022_687_MOESM3_ESM.pdf]

Description of Additional Supplementary Files

File name: Supplementary Movie 1

Description: Melting of colloidal crystal triggered by an external magnetic field of strength H = 550 A m-1. Timestamp represents HR:MIN:SEC.

File name: Supplementary Movie 2

Description: Multiple cycles of magnetic field-driven melting (H = 1000 A m-1) and depletion-driven crystallization. Timestamp represents HR:MIN:SEC. Supplementary

File name: Supplementary Movie 3

Description: Particles arrangement in dynamic clusters recorded after 12 hours of exposure to a magnetic field of strength H = 290 A m-1. Timestamp represents HR:MIN:SEC.
